# 3CLpro inhibitors: DEL-based molecular generation

**DOI:** 10.3389/fphar.2022.1085665

**Published:** 2022-12-07

**Authors:** Feng Xiong, Honggui Xu, Mingao Yu, Xingyu Chen, Zhenmin Zhong, Yuhan Guo, Meihong Chen, Huanfang Ou, Jiaqi Wu, Anhua Xie, Jiaqi Xiong, Linlin Xu, Lanmei Zhang, Qijian Zhong, Liye Huang, Zhenwei Li, Tianyuan Zhang, Feng Jin, Xun He

**Affiliations:** ^1^ Shenzhen Innovation Center for Small Molecule Drug Discovery Co., Ltd., Shenzhen, China; ^2^ Shenzhen NewDEL Biotech Co., Ltd., Shenzhen, China

**Keywords:** del, machine learning, molecule generation, 3C-like protease, transfer learning

## Abstract

Molecular generation (MG) *via* machine learning (ML) has speeded drug structural optimization, especially for targets with a large amount of reported bioactivity data. However, molecular generation for structural optimization is often powerless for new targets. DNA-encoded library (DEL) can generate systematic, target-specific activity data, including novel targets with few or unknown activity data. Therefore, this study aims to overcome the limitation of molecular generation in the structural optimization for the new target. Firstly, we generated molecules using the structure-affinity data (2.96 million samples) for 3C-like protease (3CLpro) from our own-built DEL platform to get rid of using public databases (e.g., CHEMBL and ZINC). Subsequently, to analyze the effect of transfer learning on the positive rate of the molecule generation model, molecular docking and affinity model based on DEL data were applied to explore the enhanced impact of transfer learning on molecule generation. In addition, the generated molecules are subjected to multiple filtering, including physicochemical properties, drug-like properties, and pharmacophore evaluation, molecular docking to determine the molecules for further study and verified by molecular dynamics simulation.

## 1 Introduction

Drug structural optimization (Stokes et al., 2020; Wang Z.-Y. et al., 2022) is to design new molecules with better specific properties, either to increase desired bioactivities or decrease side effects. In the early stages, the conception and evaluation of new proposed molecules rely on medicinal chemists’ experience and knowledge of basic chemistry and biology. Later, with the improved computer-aided drug programming level, the ligand-based quantitative structure-activity relationship (QSAR) model (Cherkasov et al., 2014) combined with molecular docking and molecular dynamics simulation were used for large numbers of molecule screening to obtain molecules efficiently. With the substantial increase in data and the continuous improvement of computing resources, deep learning (DL) has developed rapidly. This new tool facilitated drug development, especially structural optimization. Zhavoronkov et al. (Zhavoronkov et al., 2019) discovered a kinase inhibitor of DDR1 in 41 days by building a deep-learning molecular generation architecture GENTRL. Then, applying deep learning in drug design became one of the top 10 breakthrough technologies in MIT Technology Review 2020 (MIT Technology Review, 2020). Full use of this tool can explore more expansive chemical space and generate molecules of desired physicochemical and pharmacological properties, accelerating drug development (Xu et al., 2019; Arús-Pous et al., 2020; Kotsias et al., 2020).

The strategy for molecular generative models through deep learning can be divided into ligand-based and structure-based (also called receptor-based). Ligand-based molecule generation requires a set of experimentally validated active compounds. ML generates molecules by learning the common features of the active compounds (Liu et al., 2021; Wang M.-Y. et al., 2022). Structure-based molecular generation considers ligand and receptor interactions. Traditional structure-based molecular generation is a fragment-based approach that adds, deletes, or replaces chemical fragments of ligands in pockets (Batool et al., 2019; Krishnan et al., 2022). The Algorithm using the protein’s structural information to design new molecules has not been widely validated due to the limitation of high computational resource consumption (Skalic et al., 2019; Born et al., 2021; Grechishnikova, 2021). A structure-based molecular generation often requires three-dimensional information on the binding pockets within ligands (Wang M.-Y. et al., 2022; Long et al., 2022). Facing new targets, often there are neither revealed binding pockets nor experimentally validated ligands. The datasets used for ligand-based molecule generation usually come from public databases (such as CHEMBLE, ZINC, etc.), and the specific targets sub-datasets are generally needed to guide the structural optimization. Such a procedure has an unavoidable limitation because of its heavily dependent on public experimental data. For new targets, such dataset is severely lacking. Machine learning cannot be developed without an available dataset. This is the main reason for molecular generation, and even AIDD is still challenging to apply to the structure optimization of hit compounds for new targets. DNA-encoded library (DEL) (Dickson et al., 2019; Li et al., 2022; Nie et al., 2022; Song et al., 2020; Yang et al., 2022; Zhao et al., 2019; Zhao et al., 2022) is a powerful tool from combinatorial screening and DNA-encoded technology. Compared with traditional high-throughput screening (HTS), DEL technology can efficiently and economically generate a large amount of affinity data for specific targets, including new target data (hundreds of billions scale) (Buller et al., 2010; Kalliokoski, 2015). Therefore, using the DEL dataset, mainly the structure-affinity relationship, for molecular generation could be a reasonable solution to the problem of efficient structural optimization for new target drug development.

Deep learning generative algorithms have been explored for aided drug design. Generally, standard inputs in generative models are linear input symbols like Simplified molecular input line entry specification (SMILES) and molecular graphs. Common generative model architectures include recurrent neural networks (RNNs) (Bjerrum and Threlfall, 2017; Segler et al., 2018; Kotsias et al., 2020), autoencoders [AE, VAE (variational AE), AAE (adversarial AE)] (Kingma and Welling, 2013; Rezende et al., 2014; Makhzani et al., 2015), generative adversarial networks (Goodfellow et al., 2020). Optimization strategies for generative models include transfer learning (Segler et al., 2018), Bayesian optimization (Gómez-Bombarelli et al., 2018), reinforcement learning (Wang et al., 2021), and conditional generation (Li et al., 2018). Transfer learning is a strategy for transferring knowledge from pre-learned tasks to improve learning performance. Public datasets are usually needed for pre-training till obtaining a greater probability of generating valid molecules. Subsequently, the pre-trained model is retrained using known active molecules. Generally, the overall distribution of the pre-trained CHEMBL or ZINC large dataset is quite different from that of specific target active molecules, negatively affecting transfer learning (Zhao et al., 2014). Transfer learning using the DEL dataset is expected to address this obstacle effectively. The DEL dataset herein is composed of 3 groups of building blocks. We used the high-affinity molecules from DEL (which appeared as compounds with high count values in DEL) to reduce the distribution inconsistency between the pre-trained model and transfer learning. The beneficial effect of transfer learning herein is confirmed and consistent with the DEL dataset. In this experiment, we used the molecular dataset with higher counts in DEL, molecules with more potent binding force to the target 3CLpro, for transfer learning, thereby increasing the probability of generating active molecules.

**SCHEME 1:**
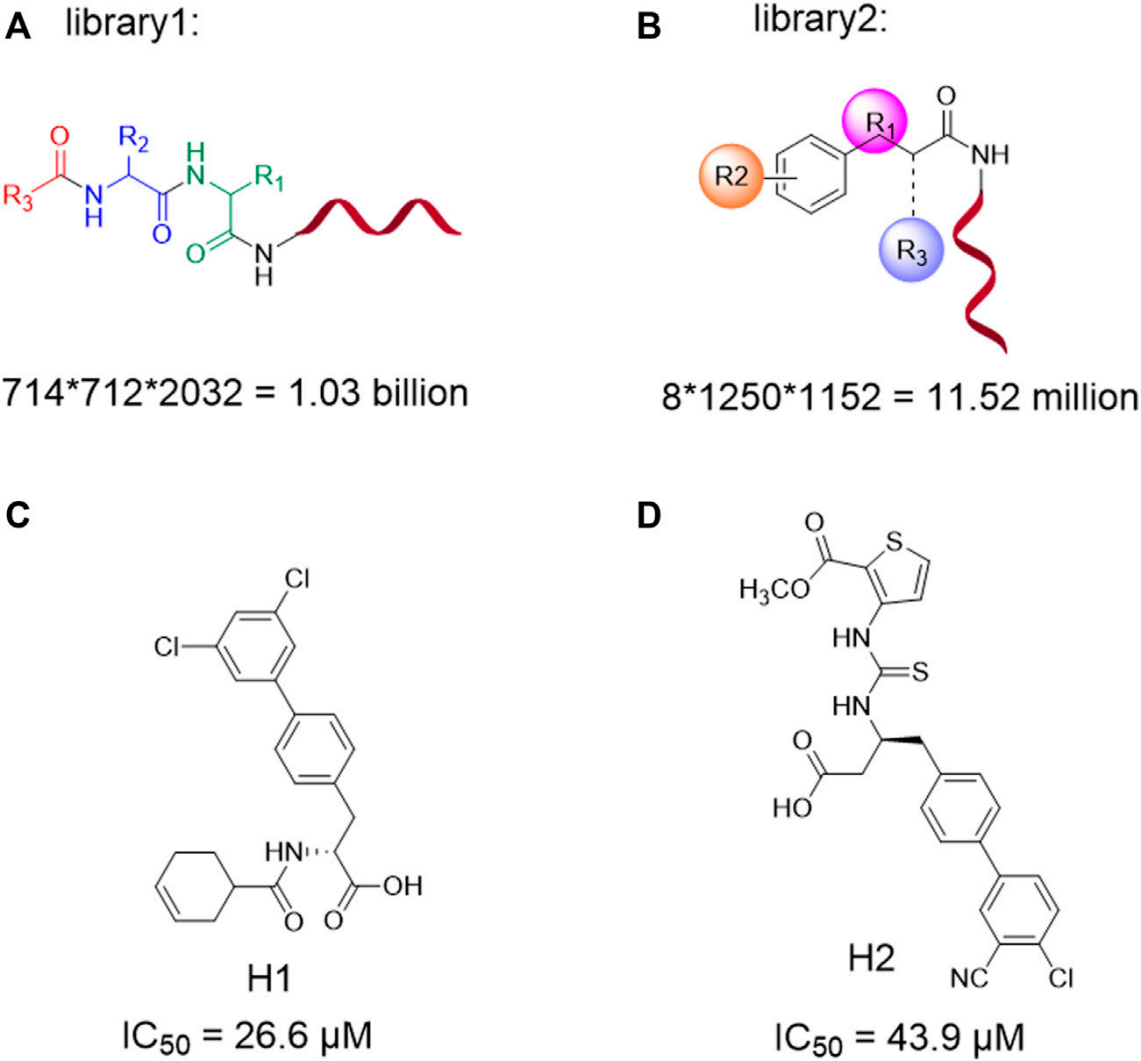
DNA-encoded library1 and library2 designed information **(A,B)**; Structures and corresponding inhibition activity for 3CLpro (IC_50_/µM) of **H1** and **H2 (C,D)**.

**FIGURE 1:**
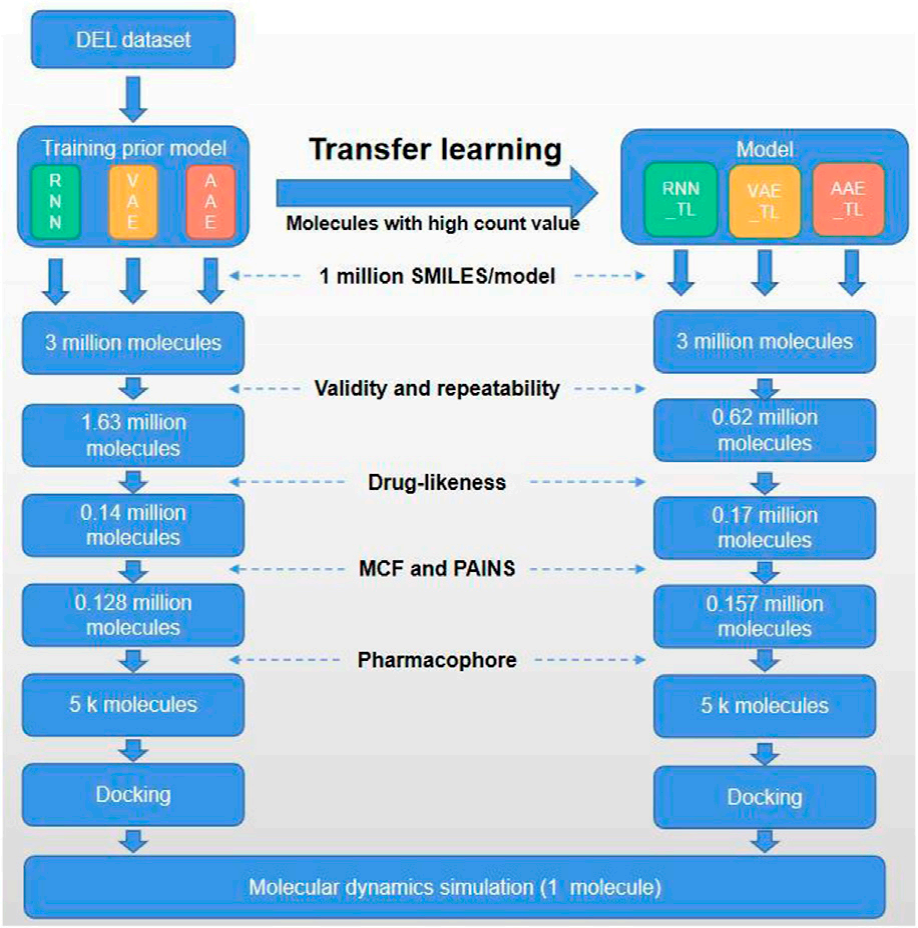
Flow chart of the current study.

In this study, we set out to solve the following challenges: using DEL technology to construct two DEL libraries for 3CLpro and performing data analysis combined with chemical synthesis. Active hit compounds **H1** and **H2** (Scheme 1) were found *via* bioactivity assay. Subsequently, the own-built DEL dataset was used to establish a molecular generative model to obtain a dataset with broad chemical space distribution. The obtained molecule dataset was directly applied with several subsequent filtering steps. On the other hand, molecules with high count values in the DEL dataset were defined as positive samples for transfer learning to obtain another dataset. The above two datasets were filtered by the druggability and pharmacophore model. Finally, the obtained molecules were verified by molecular docking and dynamics simulation, which confirmed the potential bioactivity of the newly designed molecule (Figure 1).

## 2 Materials and experiments

### 2.1 DNA-encoded libraries screening, chemical synthesis, and bio experiments

Supporting information describes DEL screening, chemical synthesis, bio-activity experiments, and compound characterization.

### 2.2 Machine learning modeling

#### 2.2.1 Data preparation

The two DEL libraries are combined as a dataset containing 1.04 billion molecules with corresponding count and enrichment fold (EF) values. We provide the information of DEL in the supplement material. We cut out the data with very low count, the remaining data is 3,702,672. Then it was divided into a training set and a test set (0.8/0.2), of which the training set and test set have 2,962,138 and 740,534 molecules, respectively. First, the training set is used to train the molecule generation model. Then, molecules 18,129) with higher count values in DEL were selected as the positive samples of transfer learning to fine-tune the pre-trained model.

#### 2.2.2 Molecular generation

SMILES or molecular graphs are commonly used for molecular representation. SMILES is a text of molecular structures (Weininger, 1988). Molecular graphs represent the structure of molecules as graphs, where the edges of the graph represent bonds and the dots represent atomic structures (Sun et al., 2020). Molecules were represented as SMILES in three models, RNN, VAE, and AAE, to perform molecule generation. We used pytorch, sklearn, numpy, rdkit,umap-learn, and MOSES benchmark platform (Polykovskiy et al., 2020) to complete the experiments.

Recurrent neural network (RNN) (Segler et al., 2018) are designed to learn sequential data such as text or speech. The SMILES in DEL are made into a corpus. RNN can learn the grammatical information about the SMILES to know which parts of molecules tend to be connected. RNN can generate sequences through forward propagation (Bjerrum and Threlfall, 2017). By treating the molecule generation process as a series of steps and sampling the network at each step, generating effective molecules is highly probable and structurally similar to the training molecules. The architecture used in RNN consists of an embedding layer, three LSTM layers, and a linear layer.

Variational autoencoder (VAE) (Bowman et al., 2015) consists of an encoder and a decoder. The former encodes the input data into a latent vector, which obeys the Gaussian Distribution. The decoder restores the latent vector result to the target sample. SMILES are used as the model’s input and output to establish a VAE model. The VAE architecture consists of an embedding layer, an encoder layer, and a decoder layer. The encoder and decoder layers consist of a GRU layer and two linear layers.

Adversarial Autoencoder (AAE) (Makhzani et al., 2015) is similar to VAE principally. The difference is that based on the encoder and the decoder, a discriminator is introduced, which is responsible for distinguishing the calculated latent vector in the encoder from the real sample. The encoder and decoder are still accountable for encoding and reconstructing the data. AAE also uses SMILES as input and output. The AAE’s encoder part includes an embedding layer, an LSTM layer, and a linear layer. The decoder consists of two linear layers, one embedding layer, and one LSTM layer. The discriminator consists of two linear layers where the activation function is ELU (Clevert et al., 2015).

#### 2.2.3 Transfer learning

Transfer learning (Amabilino et al., 2020) is a fine-tuning model technique that fixes the original model’s specific parameters while others are still training and updating. This technique aims to streamline expansive chemical space in the generative model, searching for target molecules in the relatively small chemical space. The model is retrained by inputting molecules with high-count values to generate more distribution-similar molecules than those with high-count values. To AAE, we fine-tune the decoder’s last linear layer and the discriminator’s last linear layer. To VAE, the last two linear layers of the decoder’s model are fine-tuned. To RNN, we also fine-tune the last linear layer.

#### 2.2.4 Evaluation metrics

Each model generated 10,000 molecules, which were evaluated using the evaluation metrics provided in Moses (Polykovskiy et al., 2020), including valid, unique, novelty, internal diversity (IntDiv), and scaffold similarity (Scaff).

Validity is the proportion of valid molecules in the generated molecules.
Uniqueness is the proportion of molecules not duplicated in the generated molecules.
Novelty is the proportion of molecules that do not exist in the training set.
Internal diversity (IntDiv) (Benhenda, 2018) is a metric to assess the chemical variety of generated molecules. The value range is [0,1]. The higher value means higher diversity of the generated molecules.
Scaffold similarity (Scaff) represents the similarity between scaffolds in the generated set and reference dataset. The value range is [0,1]. The higher the value of Scaff, the more similar the two are.

#### 2.2.5 Filtering

The number of molecules generated by each model is 1,000,000 molecular datasets. First, the dataset was filtered for validity and reproducibility, followed by drug-likeness: 250 ≤ MW ≤ 750, logP ≤ 5, HBD ≤ 5, HBA ≤ 10, RB < 10, and 0.5 < QED. The next step was improving drugability by applying Medicinal Chemistry filters (MCFs) (Kalgutkar et al., 2005) and Pan Assay Interference Compounds (PAINS) filtering (Baell and Holloway, 2010). Without transfer learning, RNN, AAE, and VAE have no significant performance differences and predicted affinity distribution. Therefore, these datasets are merged and divided into groups depending on whether transfer learning is applied or not. 5000 molecules were obtained after pharmacophore filtration. Finally, molecular docking was employed, and molecule with higher docking score was selected for molecular dynamics simulation.

#### 2.2.6 Chemical space visualization

The remained SMILES after validity and repeatability filtration and the original 3,702,672 SMILES from the DEL dataset were transformed into Morgan fingerprints with 1024 dimensions and 2 radius (Rogers and Hahn, 2010). These fingerprints were then used to build a UMAP(Uniform manifold approximation and projection) (metric = “jaccard,” n_components = 2) model for dimensionality reduction visualization (McInnes et al., 2018).

#### 2.2.7 Affinity modeling

The model for affinity prediction was established according to our previous study (Xiong et al., 2022). First, we sorted the molecules in the DEL dataset by the count value, then oversampled the top 10,000 ranked molecules by ten times. The step was set as 0, and every other step of the remaining molecules was sampled to form a training set.

#### 2.2.8 3D conformation and pharmacophore-based screening

The 3D molecular similarity was calculated through the shape and color similarity score (SC score), which represents the pharmacophoric feature similarity (Landrum et al., 2006) and the shape similarity (Putta et al., 2005). This score was used for the previously generated dataset. The 3D similarity score is a floating point value in the range of [0, 1], with a higher value indicating higher similarity between candidate and reference molecules. The native ligand in PDB:7L13 from the RSC-PDB database was used as a reference structure (Zhang et al., 2021). 100 conformations were generated for each molecule from the dataset using the RDKit UFF (Universal Force Field) force field. The lowest energy conformation was applied for the next step.

#### 2.2.9 Molecular docking

The A-chain of the complex PDB:7L13 (resolution 2.17 Å) of 3CLpro protein was split as a docking template to obtain accurate docking results. Subsequently, the complex was preprocessed using the Protein Preparation Wizard module of the Maestro suite (version: 13.1.141, Schrödinger Inc.) with the default setting, including the addition of hydrogen and side chains, removal of water molecules, and calculation of partial charges and protonation states using the OPLS4 force field (Poltev et al., 1996). Followed by a grid generation module, a similar-sized grid box centered on the native ligand was made to determine the binding pocket. All molecules were preprocessed by the LigPrep module. The ionization states were calculated using Epik (Shelley et al., 2007) at pH = 7.0 ± 2.0. Finally, all molecules were docked into the binding pocket within the grid and evaluated using the standard precision (SP) of Glide-v9.4. The scale factor and partial charge intercept are set to 0.8 and 0.15, respectively. 1000 poses per ligand were generated for docking evaluation. Post-docking binding site analysis and generation of interaction graphs were finished using Maestro.

#### 2.2.10 Molecular dynamics simulations

Amolecular dynamics simulation was carried out to analyze further the dynamic interaction process between protein and ligand and the stability of binding status. Molecular dynamics simulation is a popular technique to study protein motion by tracking its conformational changes over time (Collier et al., 2020). Molecular interaction and visualization analysis based on SP docking results, the top-ranked molecules were used for the molecular dynamics simulation (MD-simulation) study. MD-simulation was performed using the GROMACS software package (version 2021.5) (Rakhshani et al., 2019). The AMBER14SB force field parameter was used for the protein. The ligand atomic charge was calculated using the B3LYP/6-31G* basis set. The ligand topology was computed using the GAFF2 force field parameter. The TIP3P water model was used to add Na^+^ and Cl^−^ ions to neutralize the charge. Electrostatic interactions are handled separately using the Particle Mesh Ewald (PME) and Verlet algorithms. The heavy atoms of the protein are constrained, and the energy minimization is carried out through 50,000 steps using the steepest descent method. The simulated system was equilibrated for 100 ps using a canonical ensemble (NVT) and an isothermal-isobaric ensemble (NPT). Both van der Waals and Coulomb interactions were calculated using a cutoff of 1.4 nm. Afterward, the system was run at constant temperature (300 K) and constant pressure (1 bar) for 100 ns molecular dynamics simulations with a time step of 2 fs and trajectory data saved every 5 ps. Finally, the ligand and protein complex’s root mean square deviation (RMSD, Å) at 100 ns was measured. By examining the interaction of the ligand with active site residues and the structural changes of the complex, the complexes were considered stable.

## 3 Results and discussion

### 3.1 Machine learning modeling

#### 3.1.1 Evaluation of molecular generative models

We evaluate each model’s validity, uniqueness, novelty, intDiv, and Scaff metrics (Tables 1, 2). The validity and uniqueness indicators of all models perform satisfactorily, indicating that the models can learn the grammatical information of the SMILES structure. The performance of novelty and IntDiv indicators is relatively poor, meaning that the model’s generalization ability may not be strong enough. VAE and RNN have higher Scaff values, meaning that the model can generate the same skeleton as the training set, but the ability of generating new skeleton is weak, while AAE is the opposite. In other words, the generated dataset and the training dataset had an apparent overlap. Especially after using transfer learning, Novelty’s metrics dropped further. A more complex model may be beneficial to address such a problem, so the MCMG (Multi-constraint molecular generation) model was also established (Wang et al., 2021). Unfortunately, MCMG performed relatively poorly in affinity prediction, so we decided not to analyze it further (Supplementary Figure S13).

**TABLE 1:** Performance of each model without transfer learning.

Model	Validity	Uniqueness	Novelty	IntDiv	Scaff/Test
VAE	0.9480	**0.9990**	0.6421	0.7496	**0.8998**
AAE	0.9343	0.9981	0.6394	0.7397	0.6483
RNN	**0.9994**	0.9750	0.6116	0.7646	0.8884
MCMG	0.8611	0.9980	**0.9952**	**0.7894**	0.6359

The bold values are specific values with best-performance in each column.

**TABLE 2:** Performance of each model with transfer learning.

Model	Valid	Uniqueness	Novelty	IntDiv	Scaff/Test
VAE_TL	0.9653	0.8989	0.3692	0.7461	0.6638
AAE_TL	0.8753	0.8823	**0.4793**	0.7397	0.6177
RNN_TL	**0.9780**	**0.9299**	0.3880	**0.7503**	**0.6979**

The bold values are specific values with best-performance in each column.

#### 3.1.2 Chemical space visualization

**FIGURE 2:**
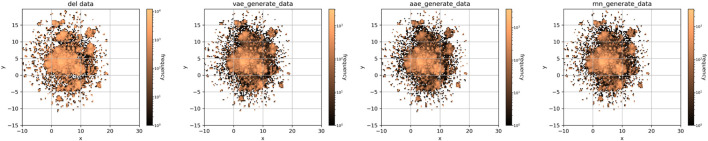
Dimensional reduction visualization of the training and generated molecular datasets (from left to right: DEL, VAE, AAE, and RNN).

The results visualization using UMAP dimensionality reduction are shown in Figure 2. The molecules generated by each model closely resemble the chemical space distribution of DEL’s. This indicates that the model could learn molecular distribution sufficiently from the source dataset.

#### 3.1.3 Affinity model performance

**FIGURE 3:**
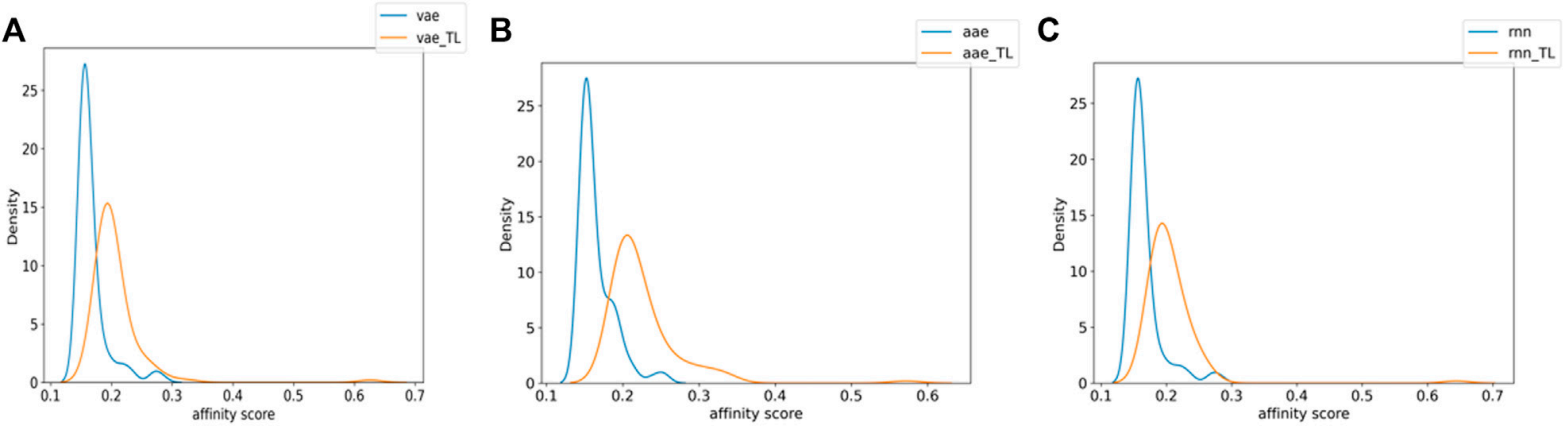
The numerical affinity distribution of molecules generated by each model with or without transfer learning [**(A)** VAE, **(B)** AAE, **(C)** RNN].

**SCHEME 2:**
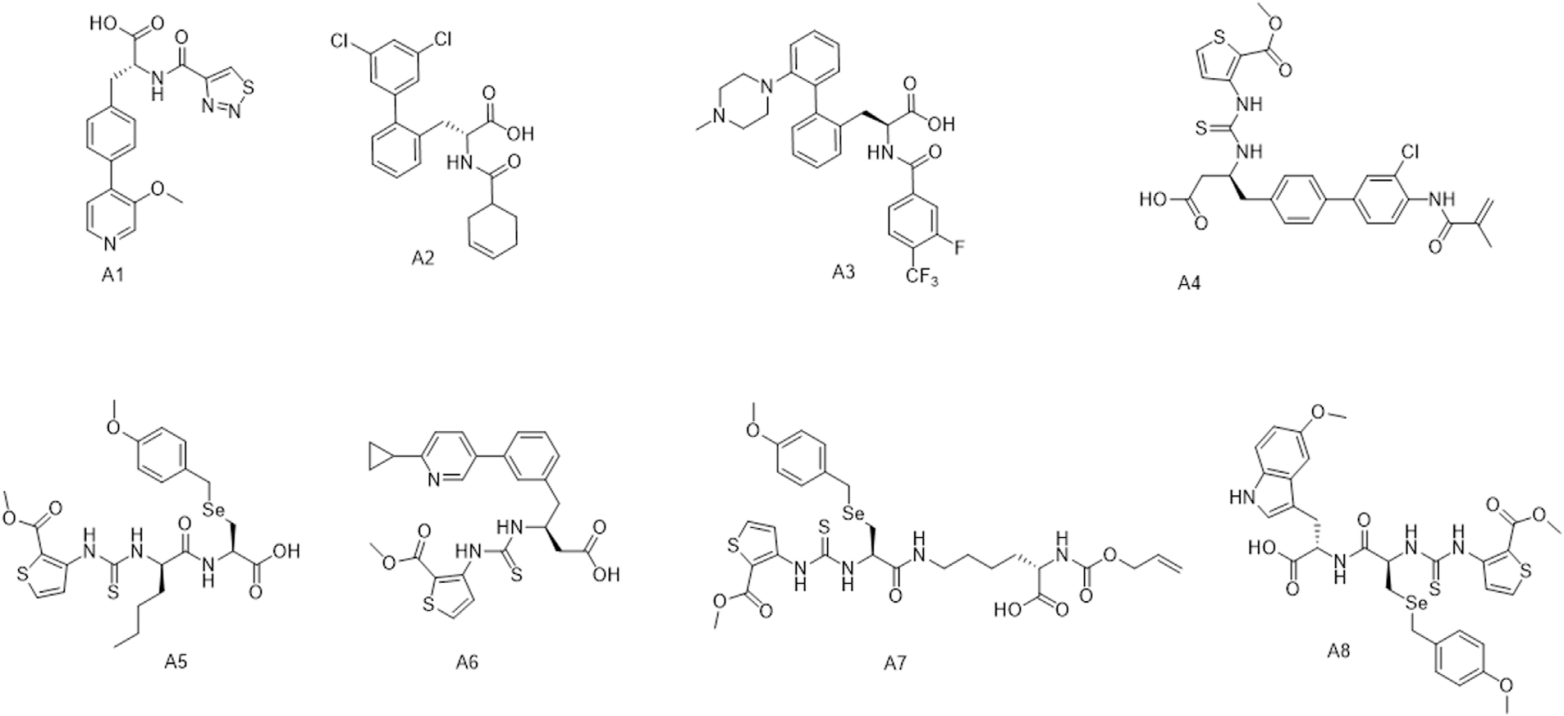
The representative molecules (**A1–A8**) with high affinity scores according to affinity model.

Figure 3 shows the distribution of the molecules from each model’s affinity predictions. The molecules distribution without transfer learning is mainly located in the area of 0.1–0.2, while the corresponding values after using transfer learning are mostly above 0.2. Such improved affinity indicates the beneficial effect of transfer learning, which expectedly to improve the success rate and efficiency for further structural optimization. Scheme 2 shows the representative molecules with high affinity scores, which were expected to be potentially bio-active.

#### 3.1.4 Molecular docking

According to the calculated SC scores of all molecules in the dataset and native ligand, 5,000 ligands with the highest SC score were selected for the follow-up study.

Molecular docking was utilized to analyze the 3D conformational and pharmacophore-based screens and to study the structural basis of the interaction between 3CLPro and ligands. First, the reliability of the glide docking algorithm (standard precision mode, enhanced conformational sampling by four times) was confirmed by re-docking the native ligand to the receptor.

The re-docked conformation was presented in supporting information. Subsequently, the selected 5000 molecules were preliminarily docked to the revealed binding site using standard precision mode. According to the docking evaluation score and molecular conformation, 500 different conformations were selected, and four times enhanced conformational sampling was used to generate the ligand-binding pose more accurately.

In addition, the docking scores with and without transfer learning for pharmacophoric models were analyzed (Table 3). Applying transfer learning, 4.9% of ligands possess a score greater than 8, while 0.3% have a score higher than 9. In contrast, the corresponding values without transfer learning are 3.3% and 0.1%. This result is consistent with the affinity model, indicating that transfer learning can effectively increase the percentage of positive samples.

**TABLE 3:** Differences in docking scores for molecules generated with and without transfer learning.

Docking scores	Model with TL (pharmacophore)	Model without TL (pharmacophore)	Model with TL (DEL-built affinity)
1 (%)-5	8.26	9.08	8.97
5-7	61.61	66.32	64.37
7-8	25.22	21.30	21.40
8-9	4.64	3.18	4.57
9-11	0.27	0.12	0.70

Moreover, the molecules filtered by the affinity model also performed molecular docking as a pharmacophoric model. This aims to explore the possibility of replacing an external pharmacophore model with an own-built affinity model. The docking scores of the filtered molecules were combined in Table 3 for comparison. We found an exciting revelation that the proportion of molecules filtered by the affinity model with docking scores of 9–11 was twice as high as that from the pharmacophore model. Therefore, replacing pharmacophore filtering with affinity models alone may be a promising option to utilize DEL’s datasets more fully.

#### 3.1.5 Binding free energy calculation

**SCHEME 3:**
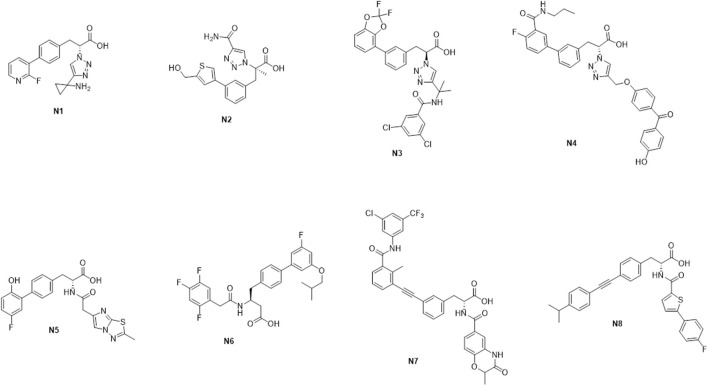
The representative molecules **N1–N8**.

The binding free energy can be used as a reference standard for assessing the activity of molecules. Generally, the lower the binding value means, the more stable the complex formed is. The ligands’ binding free energies were calculated by psp-v6.7 MMGBSA. The self-established ADMET prediction model and Maestro’s QikProp module evaluated the corresponding properties of molecules with better conformation. In fact, our ADMET prediction model is similar to ADMETlab (Dong et al., 2018). Molecules **N1-N8** were finally selected for follow-up research considering the above ADMET properties with results of binding energy calculation (Scheme 3).

**FIGURE 4:**
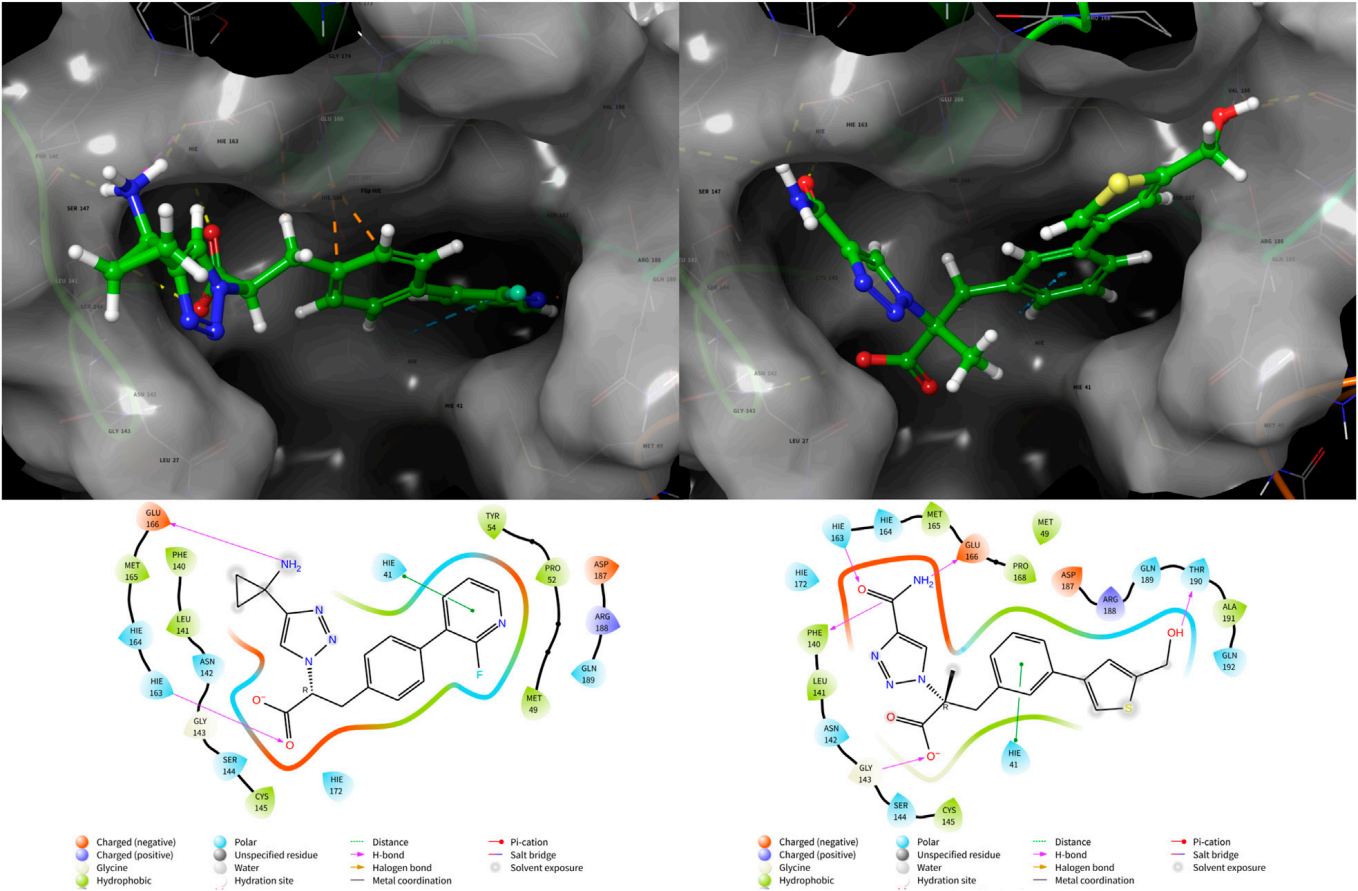
Conformation and interaction of **N1** (left) and **N2** (right) binding to 3CLPro.

From Table 4, the binding free energies (dG_Bind) of **N1** to **N8** indicate their potential biological activities. Van der Waals energy (dG_Bind_vdW) shows that hydrophobic interaction is the main contributor to the ligand binding process. According to the molecular docking conformation, we selected **N1** and **N2** for subsequent research, in which the position and interaction of **N1** and **N2** on receptors are consistent with the previous report (Figure 4) (Zhang et al., 2021; Stille et al., 2022).

**TABLE 4:** The calculated binding energy of **N1-N8** binding to 3CLPro.

Name	MMGBSA_dG_Bind	MMGBSA_dG_Bind_vdW
**N1**	−40.17 kcal/mol	−38.68 kcal/mol
**N2**	−42.12 kcal/mol	−41.44 kcal/mol
**N3**	−49.24 kcal/mol	−57.68 kcal/mol
**N4**	−50.44 kcal/mol	−60.25 kcal/mol
**N5**	−42.63 kcal/mol	−48.83 kcal/mol
**N6**	−41.25 kcal/mol	−49.25 kcal/mol
**N7**	−45.96 kcal/mol	−58.05 kcal/mol
**N8**	−34.71 kcal/mol	−51.34 kcal/mol

#### 3.1.6 Molecular dynamics simulation

To further analyze the interaction-related atomic details between molecules and 3CLPro, GROMACS was applied for molecular dynamics simulations using the above docking results. 100 ns run time for MD simulation is considered sufficient for side chain rearrangement. The result will confirm whether or not the complex remains in the most stable association. Since **N2** is believed to be more compatible with the receptor pocket (ligand conformation), and the calculation of MMGBSA binding free energy indicates that **N2** has a stronger interaction with the receptor, molecular dynamics simulations of the complex formed by **N2** were conducted.

**FIGURE 5:**
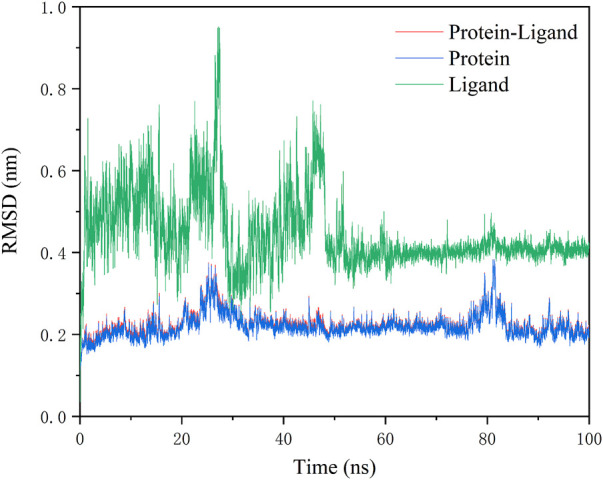
Fluctuation of RMSD values for complexes during 100 ns MD simulation.

**FIGURE 6:**
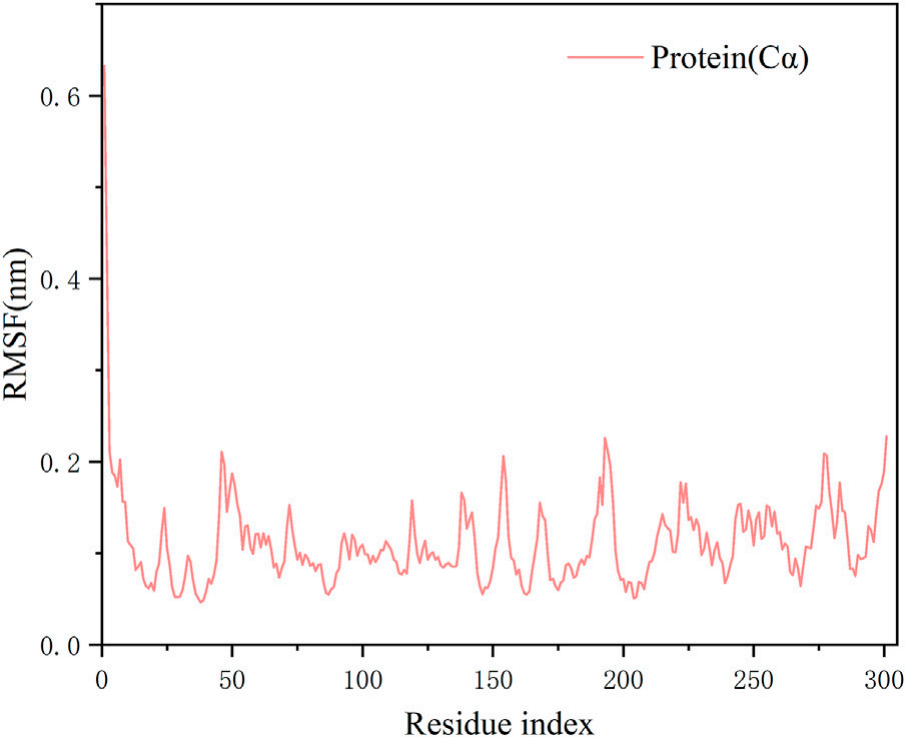
Residue-based fluctuations of protease backbone of complexes over 100 ns simulation.

RMSD values plotted over the simulation time revealed a stable kinetic equilibrium of the complex. In detail, the 3CLPro protein with **N2** and the ligand **N2** showed steady kinetics after 30 and 50 ns, respectively (Figure 5). By monitoring the fluctuation of RMSD, each system is in the range of 2Å after 50 ns. This suggests that the complex undergoes a conformational change during the simulation that promotes tight binding between the **N2** and receptor, and finally, the system reaches a steady state. The RMSF value showed minimal fluctuation, and it remained in the range of 0.05–0.2 nm throughout the simulation period for most residues, except that a peak in RMSF value was observed only at residue 1. The less fluctuating performance confirms the strong attachment of the ligand to the protein (Figure 6).

## 4 Conclusion

As far as we know, this is the first study that DEL’s dataset has been used for the molecular generation, which will promote the development of the application field of DEL combined with AI. This study preliminarily found that it may be feasible to use DEL data instead of public databases for molecular generation. In particular, for the new target pipeline, molecular generation and affinity model establishment based on DEL data are expected to become a tool with dual functions of drug discovery and further structural optimization. This advantage would be difficult to achieve with public databases due to the scarcity of datasets.

## Data Availability

The original contributions presented in the study are included in the article/Supplementary Material, further inquiries can be directed to the corresponding authors.
