# Supplementary material for: 3CLpro inhibitors: DEL-based molecular generation
Source: Front Pharmacol. 2022 Dec 7;13:1085665. doi: 10.3389/fphar.2022.1085665 (PMC9768338; doi:10.3389/fphar.2022.1085665)
Supplement: Supplementary file 1 [file Table1.DOCX]

Supplementary Material

## DNA-encoded library screening against 3CLpro

15 μL NHS beads NHS-activated Sepharose^TM^ 4 Fast Flow was washed twice with immobilization buffer (0.2 M NaHCO_3_, 0.5 M NaCl, pH 8.3) before immobilization. 200 pmol 3CLpro was diluted to 100 μL with immobilization buffer, followed by incubation with NHS beads at 4 ℃ for 16 hrs. After incubation, excessive NHS groups were quenched by 0.1 M Tris-HCl, pH 8.5 at 4 ℃ for 4 hrs. The beads were then washed with 0.1 M Tris-HCl, pH 8.5 for 3 times and 0.1 M NaOAc, 0.5 M NaCl, pH 4.5 for 3 times. The washing step was repeated for another 5 times to ensure the removal of un-immobilized proteins.

5 nmol library 1 or 200 pmol library 2 was diluted into 100 μL total volume with PBS supplemented with 0.01 mg/mL Herring Sperm DNA before selection. The 3CLpro immobilized beads were rinsed twice with PBS and incubated with library at 4 ℃ for 4 hrs. After incubation, beads were washed with PBS for 10 times to remove unbound molecules, followed by elution of remained library with 20 μL ddH_2_O at 95℃ for 20 min. The elution was subjected to PCR amplification directly and the resulting products were submitted for Illumina sequencing.


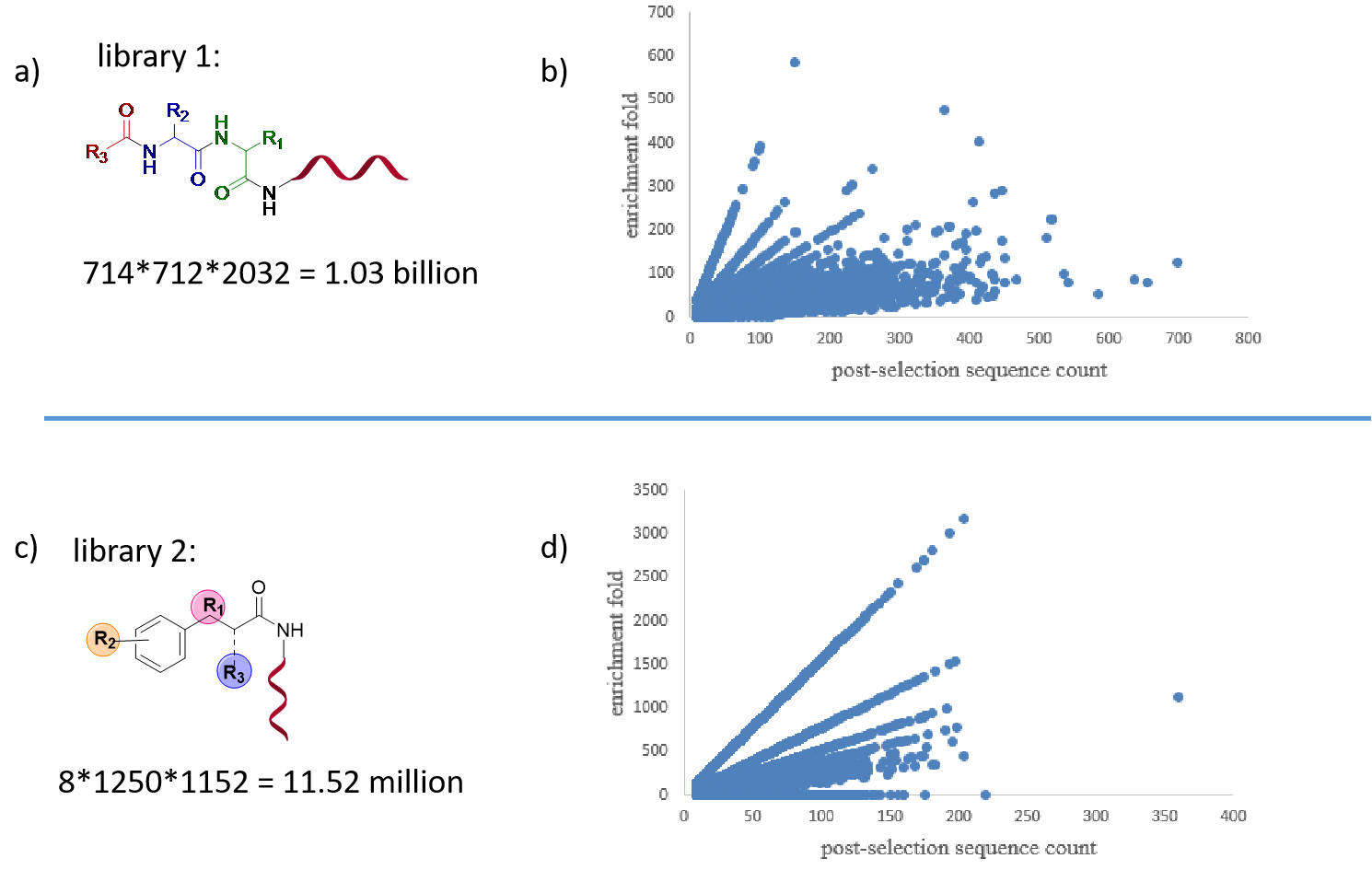


Figure **S1.** a) The structure of 1.03-billion compound. b) Two-dimensional display of post-selection DNA sequencing data from 3CL protease selection. x-axis: post-selection sequence count; y-axis: post-selection enrichment fold = (post-selection counts%/)/(pre-selection counts%); c) The structure of 11.52-million compound. d) Two-dimensional display of post-selection DNA sequencing data from 3CL protease selection. x-axis: post-selection sequence count; y-axis: post-selection enrichment fold = (post-selection counts%/)/(pre-selection counts%).

## Inhibition on 3CL protease assay

2.1 Reagent, consumables and instruments

| **Reagent** | **Vendor** | **Cat No.** |
| --- | --- | --- |
| 3CL substrate | Nanjing Peptide Biotech Ltd. | / |
| 3CL protease | R&D | E-720-050 |
| PF-07321332 | MCE | HY-138687 |
| **Consumables** | **Vendor** | **Cat No.** |
| 96 Well Plates | Nunc | 249944 |
| 384-well plate | Corning | 4514 |
| **Instrument** | **Vendor** | **Cat No.** |
| ECHO®655 SYSTEM | LABCYTE | 655 |
| Centrifuge | CENCE | TDZ5-WS |
| Microplate Reader | BMG | CLARIOstar |

- 1. Compound information and treatment

| **Entry** | **Compound ID** | **Stock Conc. (mM)** | **Start Conc. (nM)** | **Dilution folds** |
| --- | --- | --- | --- | --- |
| 1 | PF-07321332 | 10 | 1000 | 3 |
| 2 | NDDP-067 | 50 | 500000 | 4 |

2.3 Experiment procedure

| 1. Add 12 μL cmpd to 384-well dilution plate |
| --- |
| 1. Dilute cpd 1:3 or 1:4 in succession in DMSO for each column for 10 pts (refer to dilution plate map) |
| 1. Transfer 0.1 μL diluted cmpd solution in each row to 384 assay plate using Echo, each column containing 2 replicates (refer to assay plate map) |
| 1. Add 5 μL enzyme working solution to 384-well assay plate, centrifuge 1000 prm for 1 min |
| 1. Incubate at 25℃ for 10 min |
| 1. Add 5 μL substrate working solution to initiate reaction |
| 1. Incubate at 25℃ for 2 h |
| 1. Reading Ex 350 nm and Em 460 nm fluorescence signals with BMG |

2.4 Data analysis

| 1. Percent inhibition(% inh) for compound well = 100*(ave High control - cpd well)/(ave High control - ave Low control) |
| --- |
| 1. Assay robustness check with Low control and High control data:   S/B =ave High control/ave Low control,  CV%(Low control)=100* (SD Low control/ave Low control)  CV%(High control)=100*( SD High control/ave High control)  Z’=1-3*(SD Low control+ SD High control)/(ave High control- ave Low control) |
| 1. Fit the cpd IC_50_ from non-linear regression equation by XLfit 5.5.0   Y=Bottom + (Top-Bottom)/(1+10^((LogIC_50_-X)*HillSlope))  X: Log of cpd concentration  Y: Percent inhibition(% inh)  Top and Bottom: Plateaus in same units as Y  logIC_50_: same log units as X  HillSlope: Slope factor or Hill slope |

2.5 Result


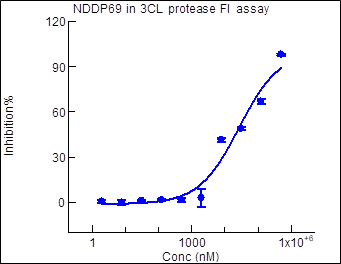

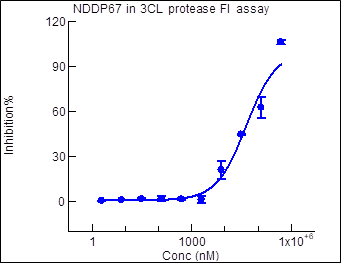


Figure **S2.** Inhibition effects on 3CL protease for hit **H1** (left). and **H2** (right).

1. **Synthesis and characterization of new compounds**

**3.1** Compound **1**

A mixture of compound **1-b** (3.00 g, 23.7 mmol), (COCl)_2_ (3.62 g, 28.5 mmol) and DMF (1.74 g, 23.7 mmol) in DCM (30.0 mL) was stirred at 0 °C for 3 hrs. TLC (petroleum ether/ethyl acetate = 1/1, R_f_=0.5) showed the reaction was complete. The reaction mixture was concentrated to give compound **1-a** (3.44 g, crude) as yellow oil.

A mixture of compound **1** (1.80 g, 7.37 mmol), compound **1-a** (3.41 g, 23.6 mmol) and TEA (1.12 g, 11.0 mmol) in DCM (20.0 mL) was stirred at 25 °C for 12 hrs. TLC (dichloromethane/methanol = 10/1, R_f_ = 0.40) showed the reaction was complete. The reaction mixture was poured into water (50.0 mL), extracted with dichloromethane (20.0 mL x 3), the organic layer was washed with sodium bicarbonate in water (acidic with hydrochloric acid). The combined organic layer and dried over Na_2_SO_4_, filter and concentrated to give compound **2** (1.27 g, 43.4% yield) as a yellow oil. ^1^H NMR: 400 MHz DMSO-*d_6_*, *δ* = 7.47 (d, *J* = 8.0 Hz, 2 H) 7.19 (d, *J* = 8.0 Hz, 2 H) 5.63 (s, 2 H) 4.46 *-* 4.37 (m, 1 H) 3.08*-* 2.99 (m, 1 H) 2.91*-* 2.79 (m, 1 H) 2.37 *-* 2.28 (m, 2 H) 2.27 - 2.20 (m, 2 H) 2.18 - 2.10 (m, 2 H) 1.68 *-* 1.76 (m, 1 H).

To a solution of K_2_CO_3_ (235 mg, 1.70 mmol) in H_2_O (1.50 mL), compound **2** (0.300 g, 851 μmol) and compound **3** (487 mg, 2.56 mmol) in dioxane (6.00 mL) was added Pd(PPh_3_)_4_ (98.4 mg, 85.1 μmol) drop wise, then stirred 90 °C for 6 hrs under N_2_. After cooling down to rt, the reaction mixture was poured into water (20.0 mL), extracted with ethyl acetate (20.0 mL x 2). The aqueous phase was adjusted to acid with 2M hydrochloric acid solution, extracted with ethyl acetate (20.0 mL x 2), dried over Na_2_SO_4_, filtered and concentrated to give a residue. The residue was purified by prep-HPLC (column: Phenomenex luna C_18_ 150 x 25 mm x 10 μm; mobile phase: [water (FA)-ACN];B%: 52%-82%,8min) to give **H1** (106.48 mg, 22.4% yield) as yellow solid. LCMS: m/z = 418.2 (M+H^+^), HRMS(ESI) C22H22O3NCl2 Counted:418.0971, Found:418.0972.

^1^H NMR: 400 MHz MeOD, *δ* = 7.59 *-* 7.54 (m, 4 H) 7.42 (t, *J* = 2.0 Hz, 1 H) 7.37 - 7.34 (m, 2 H) 5.66 (s, 2 H) 4.74 - 4.72 (m, 1 H) 3.31 *-* 3.28 (m, 1 H) 3.04 - 3.01 (m, 1 H) 2.49 *-* 2.40 (m, 1 H) 2.11 *-* 2.03 (m, 2 H) 1.89 *-* 1.83 (m, 1 H) 1.70 (s, 2 H) 1.36 *-* 1.28 (m, 1 H). ^13^C NMR (101 MHz, DMSO) δ 174.99, 173.22, 143.45, 138.69, 135.20, 134.68, 129.94, 126.72, 126.66, 126.41, 126.32, 125.78, 125.75, 125.17, 53.12, 40.43, 36.36, 27.56, 27.38, 25.46, 25.31, 24.29, 24.25.

Compound **3** (600 mg, 1.67 mmol, 1.00 eq), compound **4** (360.00 mg, 1.98 mmol, 1.18 eq) , Pd(dppf)Cl_2_•CH_2_Cl_2_ (180.00 mg, 220.42 μmol, 0.13 eq), Cs_2_CO_3_ (1.68 g, 5.16 mmol, 3.08 eq) was added in dioxane (6.00 mL) and H_2_O (1.50 mL) at 25 °C, then the reaction mixture was stirred at 80 °C for 12 hrs. After cooling down, the reaction mixture was poured into H_2_O (30.0 mL, pH=9), and extracted with EA (30.0 mL x 3), and then the organic layer was discarded. The aqueous phase was adjusted to pH=2-3 with 1N HCl and extracted with EA (30.0 mL x 3). The combined organic layer dried over Na_2_SO_4_, filtered and concentrated. Compound **5** (570 mg, 70.54% yield, 86% purity) was obtained as black brown solid. LCMS: m/z = 437.2 (M+Na^+^).

To a solution of compound **5** (300 mg, 723.10 μmol, 1.00 eq) in DCM (4.00 mL) was added HCl/dioxane (4 M, 5 mL, 27.66 eq) at 25 °C. After stirring at 25 °C for 4 hrs, the mixture was concentrated to give compound **6** (230 mg, crude, HCl) as black brown solid. LCMS: m/z = 315.2 (M+H^+^). ^1^H NMR: (400 MHz, DMSO-d6), *δ* = 13.0 - 12.5 (m, 1H), 8.33 (d, *J* = 2.4 Hz, 1H), 8.08 - 8.05 (m, 1H), 7.86 - 7.76 (m, 3H), 7.43 - 7.37 (m, 2H), 3.57 (s, 2H), 3.09 - 2.83 (m, 3H), 2.58 - 2.53 (m, 2H).

To a solution of compound **7** (200 mg, 1.27 mmol, 1.00 eq) in DCM (2.00 mL), then SCDI (400 mg, 2.27 mmol, 1.79 eq) in DCM (3.00 mL) was added dropwise at 20 °C. After stirring at 20 °C for 12 h, the reaction mixture was poured into H_2_O (20.0 mL) and extracted with EA (15.0 mL x 3). The combined organic layer was washed with brine (20.0 mL x 2), dried over Na_2_SO_4_, filtered and concentrated to give compound **8** (320 mg, crude) as yellow solid.

To a mixture of compound **6** (200 mg, 569.43 μmol, 1.00 eq, HCl) in DCM (1.00 mL) was added TEA (29.08 mg, 287.38 μmol, 40 μL, 0.5 eq) to make the reaction mixture pH to 7, then stirred at 25 °C for 20 min, then a solution of compound **8** (114 mg, 572.15 μmol, 1.00 eq) in DCM (1.00 mL) was added to the reaction at 25 °C. After stirring at 25 °C for 20 min, the reaction mixture was added AcOH to make pH to 5-6, then concentrated at 30 °C-35 °C. The crude product was purified by Pre-HPLC (column: Welch Xtimate C18 150 x 25 mm x 5 μm; mobile phase: [water(TFA)-ACN]; B%: 45%-75%,10 min) to provide **H2** (85.34 mg, 163.70 μmol, 28.75% yield, 98.6% purity) as off-white solid. LCMS: m/z = 514.2 (M+H^+^), HRMS(ESI) C_24_H_21_O_4_N_3_ClS_2_ Counted:514.0657, Found:514.0660. ^1^H NMR: 400 MHz, DMSO-d_6_, *δ* = 12.7 - 12.1 (m, 1H), 10.0 (s, 1H), 9.24 (d, *J* = 7.2 Hz, 1H), 8.42 - 8.23 (m, 2H), 8.05 (dd, *J* = 1.8, 8.4 Hz, 1H), 7.91 - 7.62 (m, 4H), 7.40 (d, *J* = 8.0 Hz, 2H), 4.98 - 4.69 (m, 1H), 3.99 - 3.74 (m, 3H), 3.12 - 2.85 (m, 4H).^13^C NMR (101 MHz, DMSO) δ 179.54, 172.72, 163.67, 145.93, 140.09, 139.48, 135.33, 134.51, 133.21, 132.74, 131.43, 130.96, 130.43, 127.32, 124.53, 116.49, 113.06, 110.98, 52.96, 52.44, 38.65, 37.75.

**3.3 ^1^H-NMR and mass spectra (MS) of compounds**


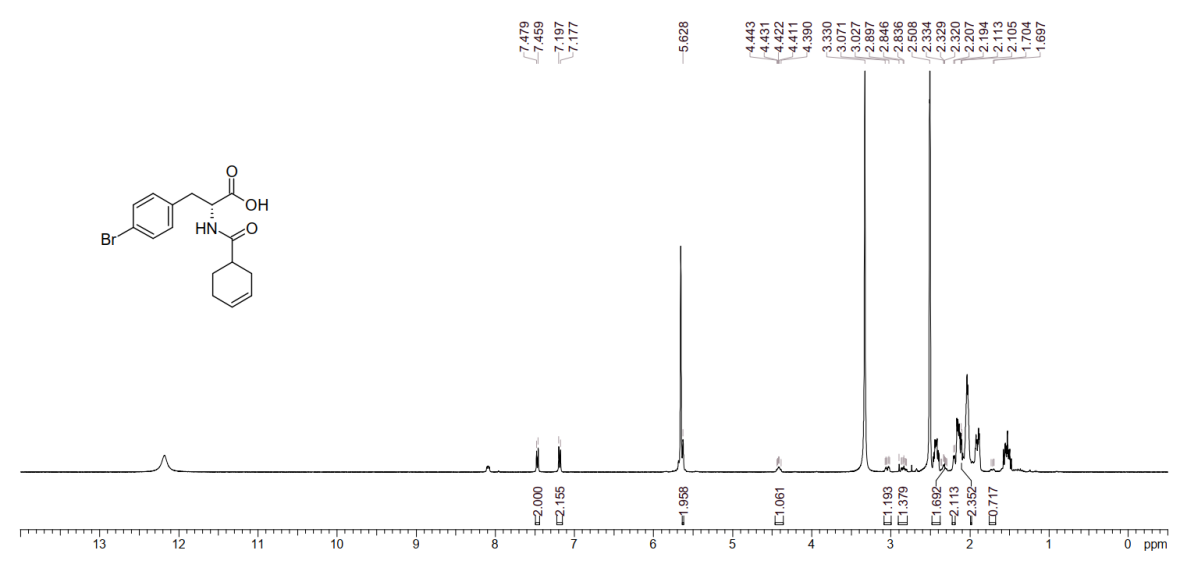


Figure **S3.** ^1^H-NMR spectrum of compound **2** in DMSO-d_6_.


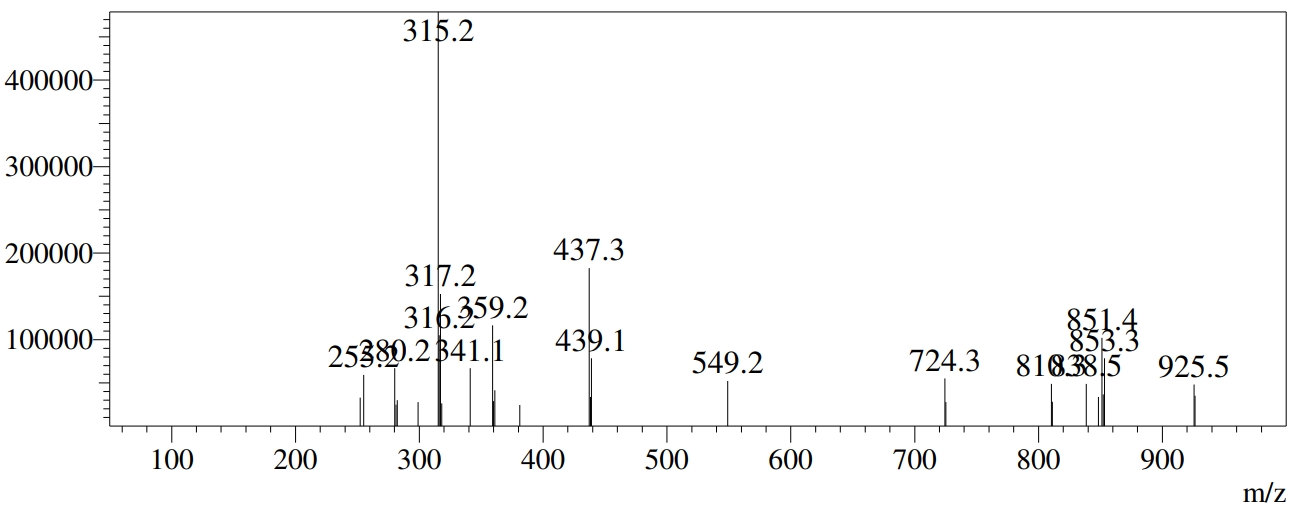


Figure **S4.** ESI mass spectrum of compound **5**.


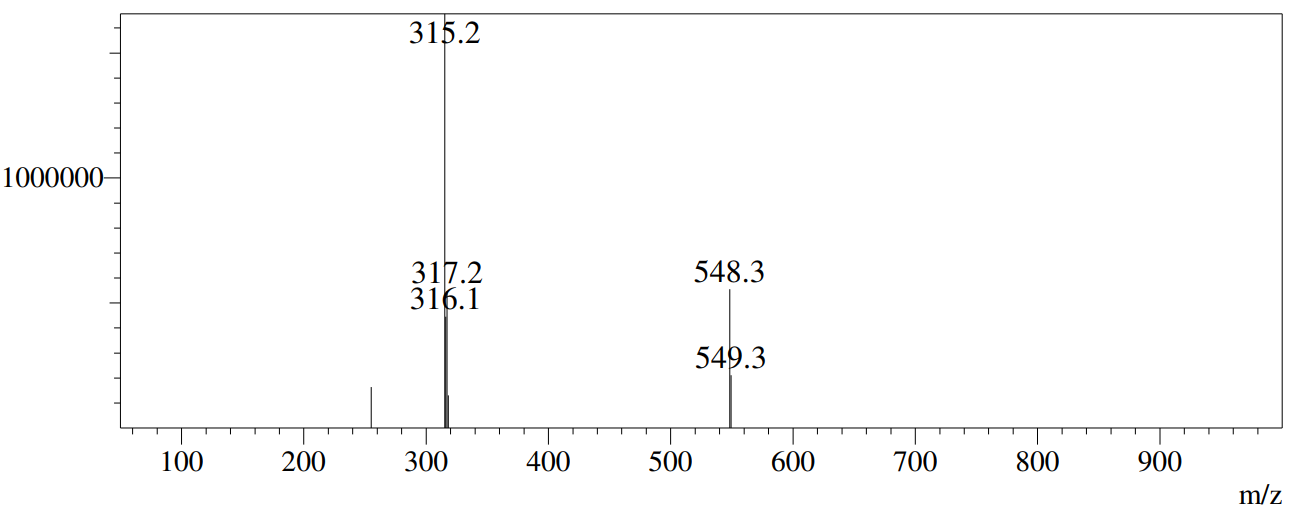


Figure **S5.** ESI mass spectrum of compound **6**.


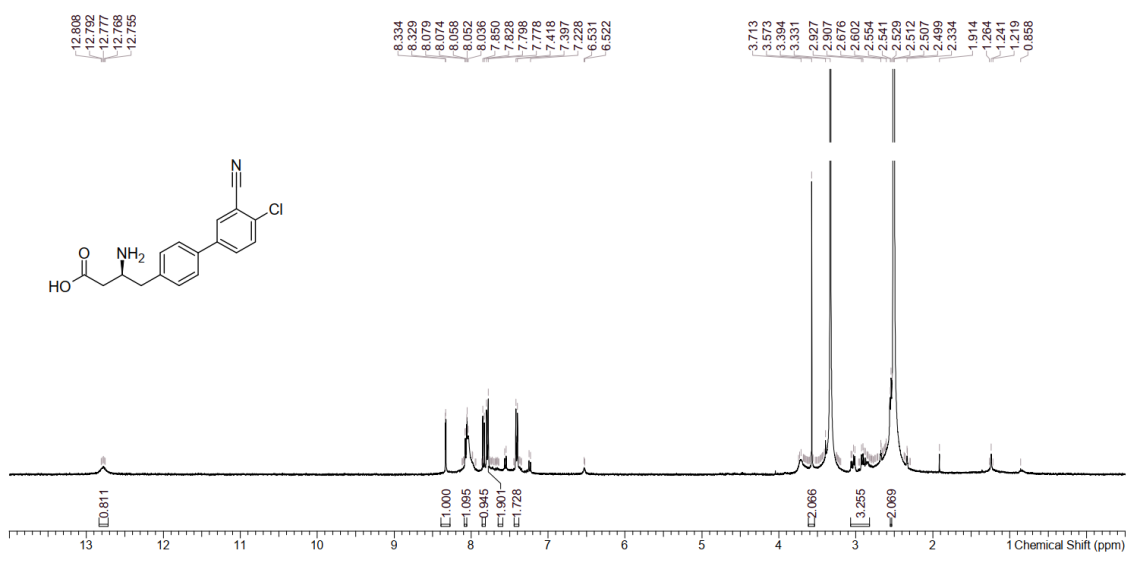


Figure **S6.** ^1^H-NMR spectrum of compound **6** in DMSO-d_6_.


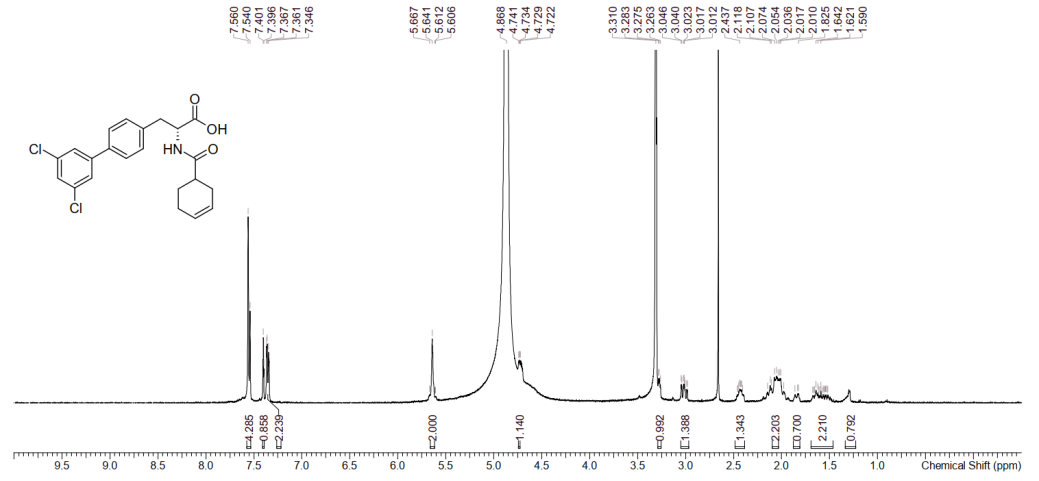


Figure **S7.** ^1^H-NMR spectrum of compound **H1** in MeOD.


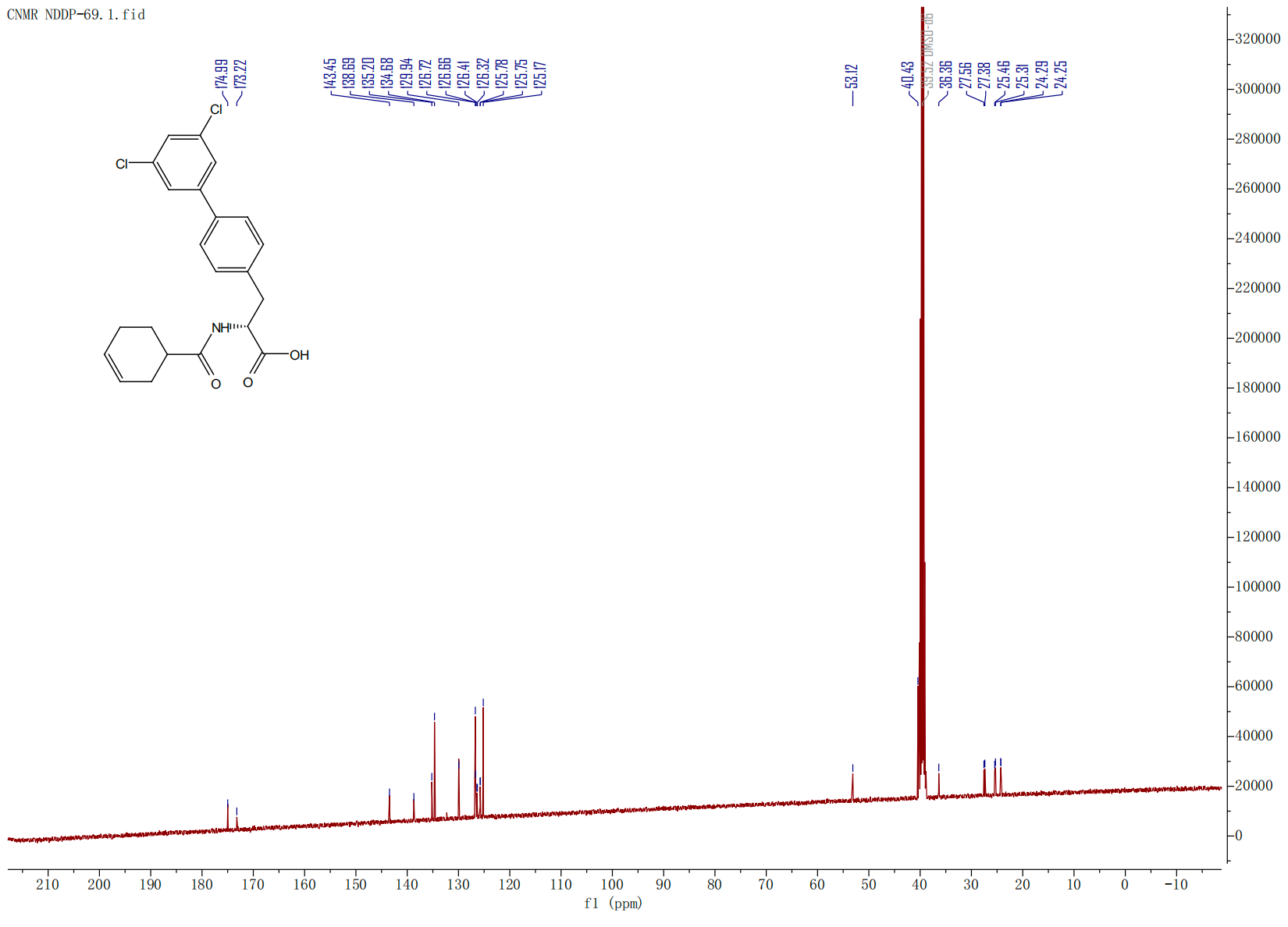


Figure **S8.** ^13^C-NMR spectrum of compound **H1** in MeOD.


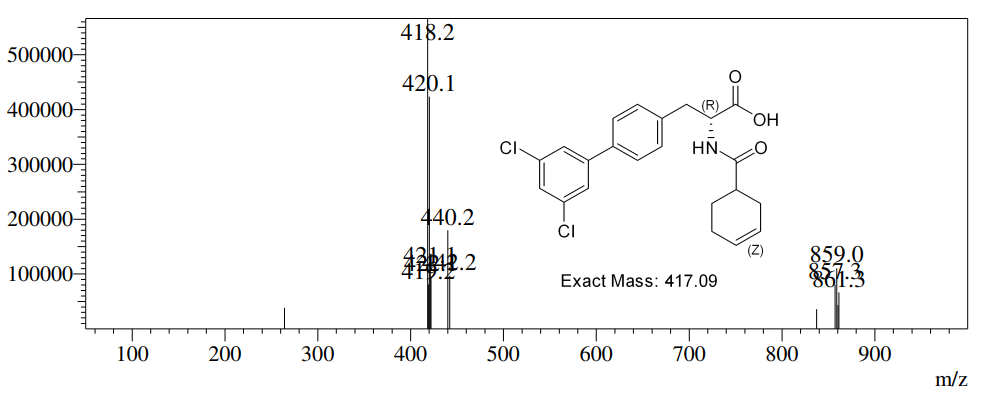


Figure **S9.** ESI mass spectrum of compound **H1**.


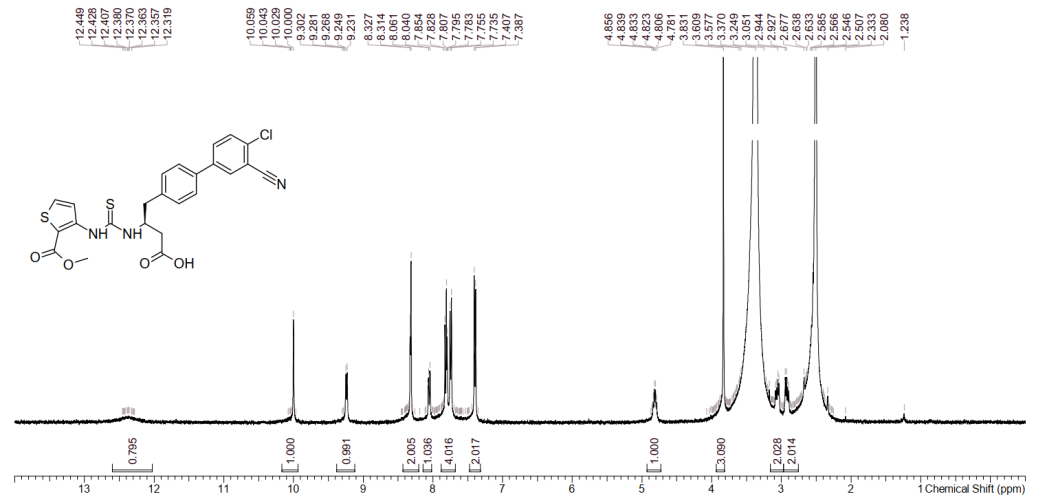


Figure **S10.** ^1^H-NMR spectrum of compound **H2** in DMSO-d_6_.


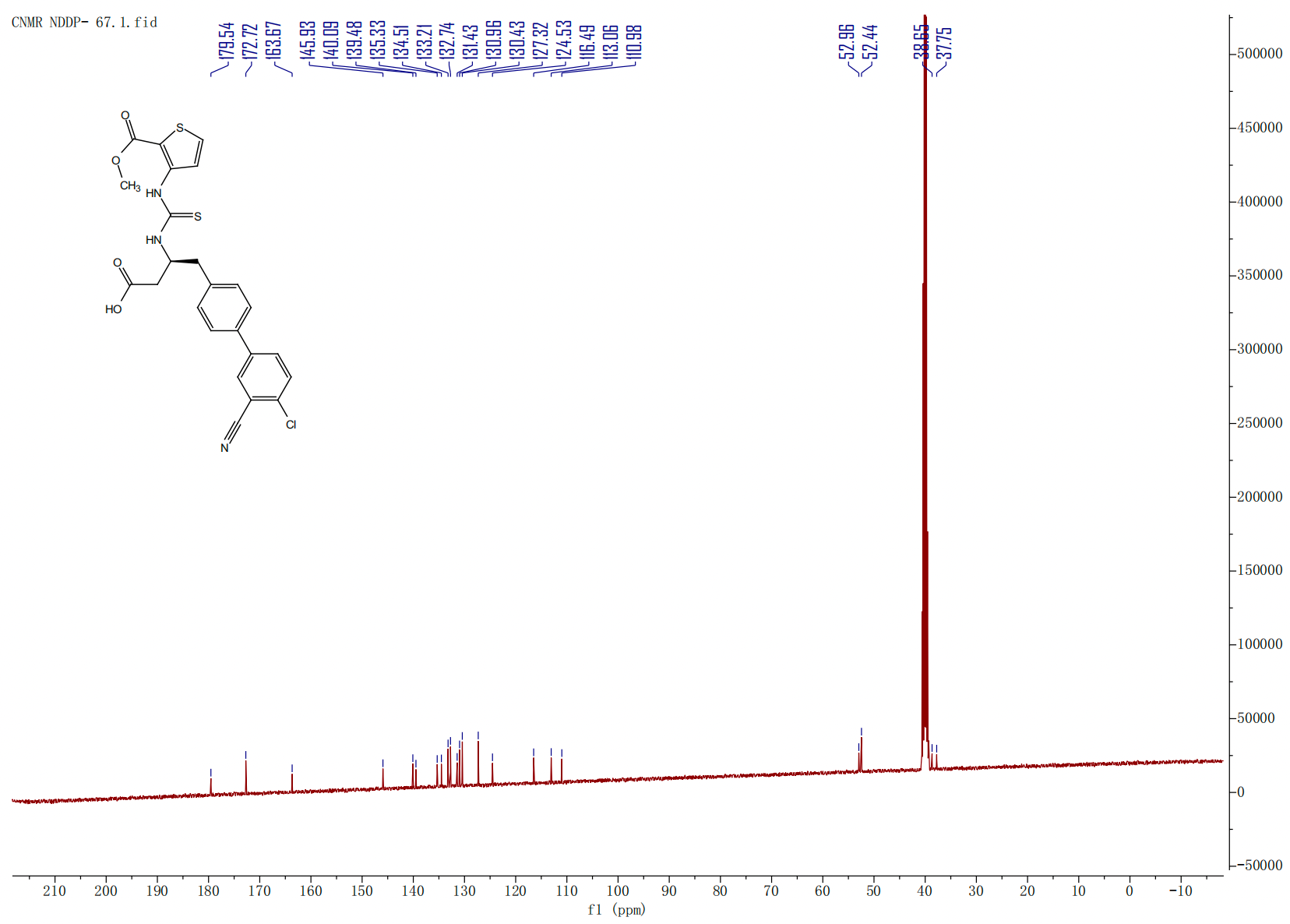


Figure **S11.** ^13^C-NMR spectrum of compound **H2** in DMSO-d_6_.


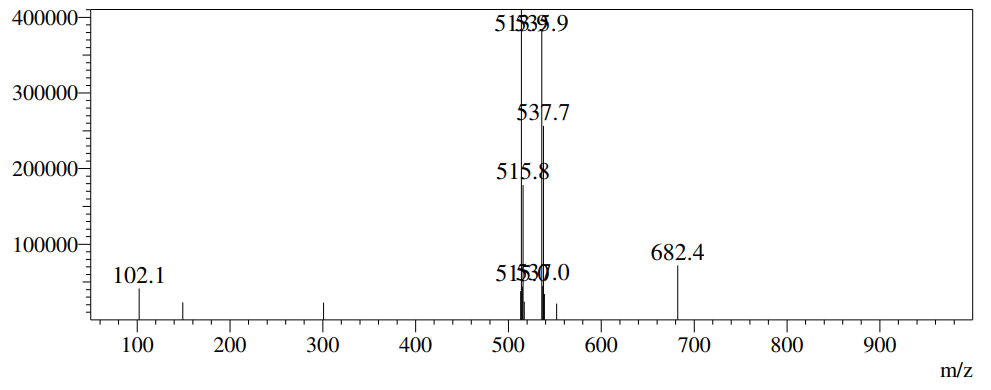


Figure **S12.** ESI mass spectrum of compound **H2**.

**
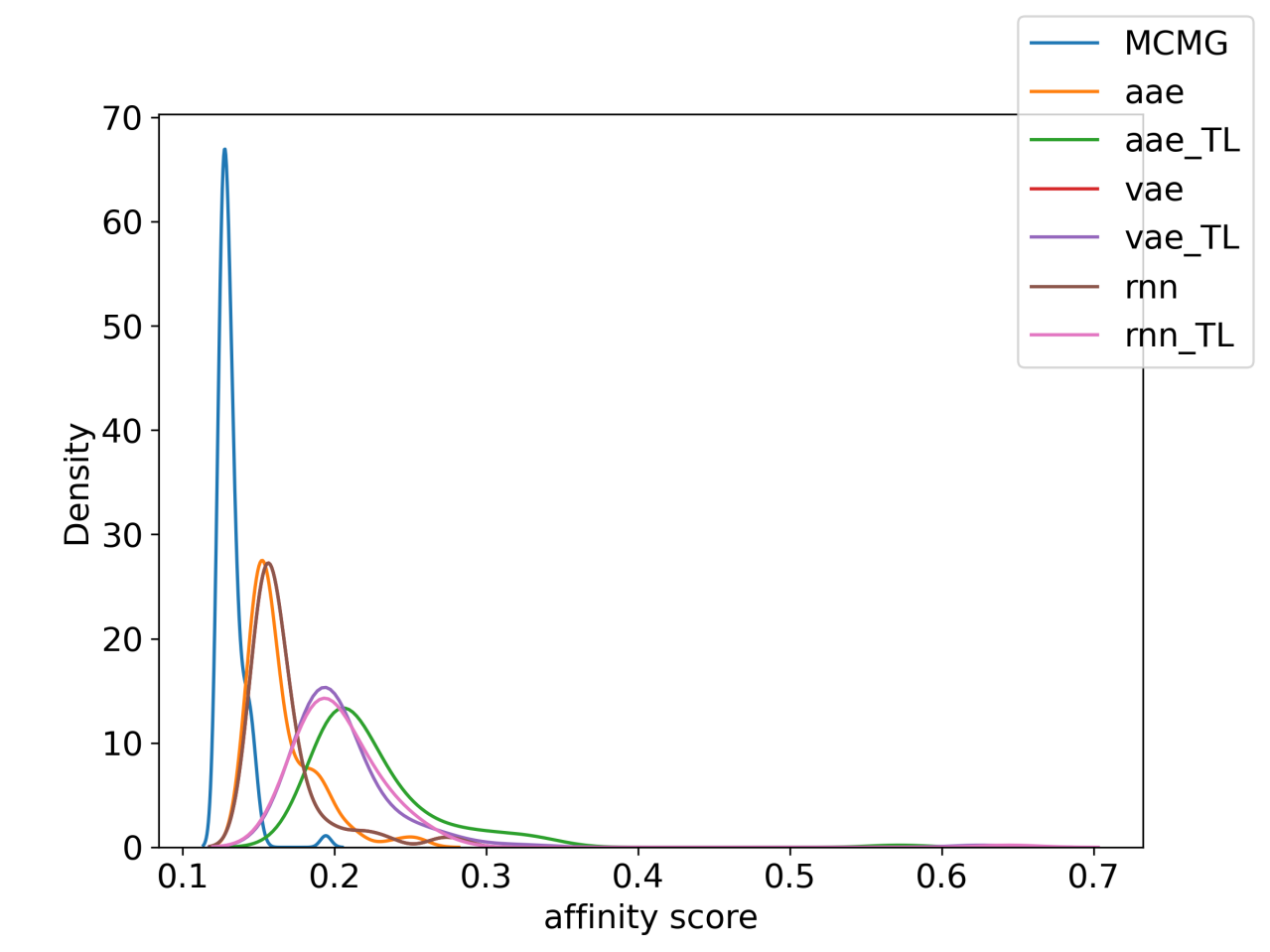
**

Figure **S13**. The numerical affinity distribution of molecules generated of each model

**
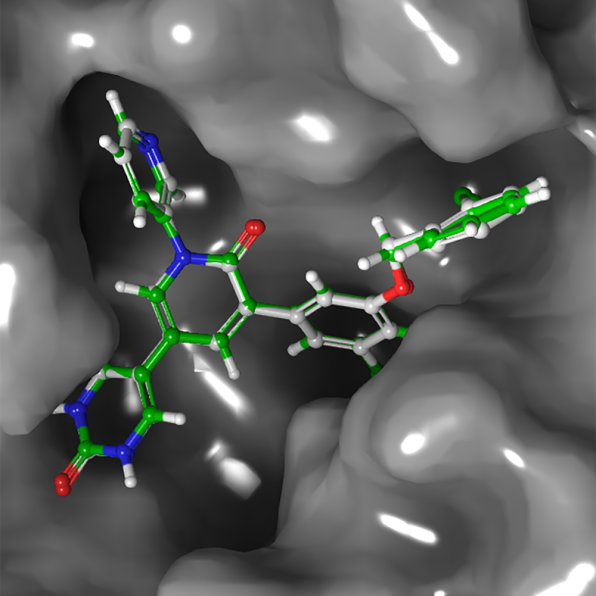
**

Figure **S14**. The surface of 3CLPro and the alignment-based docking pose of the native ligand. White and green skeletons represent the native and the docked ligand pose.
